# Prevalence of multidrug-resistant hypervirulent *Klebsiella pneumoniae* without defined hypervirulent biomarkers in Anhui, China: a new dimension of hypervirulence

**DOI:** 10.3389/fmicb.2023.1247091

**Published:** 2023-10-05

**Authors:** Md Roushan Ali, Yu Yang, Yuanyuan Dai, Huaiwei Lu, Zhien He, Yujie Li, Baolin Sun

**Affiliations:** ^1^Department of Oncology, The First Affiliated Hospital of USTC, Division of Life Sciences and Medicine, University of Science and Technology of China, Hefei, Anhui, China; ^2^Department of Emergency Medicine, The Affiliated Provincial Hospital of Anhui Medical University, Hefei, Anhui, China; ^3^Department of Clinical Laboratory, The First Affiliated Hospital of USTC, Division of Life Sciences and Medicine, University of Science and Technology of China, Hefei, Anhui, China

**Keywords:** multidrug-resistant hypervirulent *Klebsiella pneumoniae* (MDR-hvKp), *rmpA* and *rmpA2*, hypervirulence, hypermucoviscosity, virulence factors, *wzc*, whole genome sequencing

## Abstract

*Klebsiella pneumoniae* is an opportunistic pathogen that mainly causes nosocomial infections and hospital-associated pneumonia in elderly and immunocompromised people. However, multidrug-resistant hypervirulent *K. pneumoniae* (MDR-hvKp) has emerged recently as a serious threat to global health that can infect both immunocompromised and healthy individuals. It is scientifically established that plasmid-mediated regulator of mucoid phenotype genes (*rmpA* and *rmpA2*) and other virulence factors (aerobactin and salmochelin) are mainly responsible for this phenotype. In this study, we collected 23 MDR-hvKp isolates and performed molecular typing, whole genome sequencing, comparative genomic analysis, and phenotypic experiments, including the *Galleria mellonella* infection model, to reveal its genetic and phenotypic features. Meanwhile, we discovered two MDR-hvKp isolates (22122315 and 22091569) that showed a wide range of hypervirulence and hypermucoviscosity without *rmpA* and *rmpA2* and any virulence factors. In phenotypic experiments, isolate 22122315 showed the highest hypervirulence (infection model) with significant mucoviscosity, and conversely, isolate 22091569 exhibited the highest mucoviscosity (string test) with higher virulence compared to control. These two isolates carried carbapenemase (*bla*_KPC − 2_), β-lactamase (*bla*_OXA − 1_, *bla*_TEM − 1B_), extended-spectrum β-lactamase (ESBL) genes (*bla*_CTX − M − 15_, *bla*_SHV − 106_), outer membrane protein-coding genes (*ompA*), fimbriae encoding genes (*ecpABCDER*), and enterobactin coding genes (*entAB*, *fepC*). In addition, single nucleotide polymorphism analysis indicated that both isolates, 22122315 and 22091569, were found to have novel mutations in loci *FEBNDAKP_03184* (c. 2084A > C, p. Asn695Thr), and *EOFMAFIB_02276* (c. 1930C > A, p. Pro644Thr), respectively. Finally, NCBI blast analysis suggested these mutations are located in the *wzc* of the capsule polysaccharide (*cps*) region and are responsible for putative tyrosine kinase. This study would be a strong reference for enhancing the current understanding of identifying the MDR-hvKp isolates that lacked both mucoid regulators and virulence factors.

## 1. Introduction

*Klebsiella pneumoniae* is an opportunistic pathogen that causes several infections, including pneumonia, sepsis, liver abscess, bacteremia, and meningitis (Paczosa and Mecsas, 2016; Choby et al., 2020; Heiden et al., 2020). It caused the second primary bloodstream infection among gram-negative bacteria after *Escherichia coli* (Magill et al., 2014; Martin and Bachman, 2018). Besides, the World Health Organization (WHO) recognized *K. pneumoniae* as a critical (priority-1) antimicrobial-resistant microorganism requiring novel control approaches (World Health Organization, 2017; Wyres et al., 2020).

*K. pneumoniae* is classified into two significant phenotypes based on virulence, namely classical *K. pneumoniae* (cKp) and hypervirulent *K. pneumoniae* (hvKp) (Russo and Marr, 2019; Spadar et al., 2023). The most prevalent is cKp, which causes infection in immunocompromised people and acquires antibiotic-resistance genes. In addition to cKp, hvKp with hypermucoviscous phenotype has emerged as a leading clinical pathogen, causing extremely invasive infections in both immunocompromised and healthy individuals (Prokesch et al., 2016; Rossi et al., 2018; Heiden et al., 2020). Furthermore, roughly half of all hvKp infections occur in healthy young people (Shon et al., 2013; Struve et al., 2015; Paczosa and Mecsas, 2016).

HvKp was first reported in China in 1982 with multiple site infection and hyper-mucoid phenotype followed by sporadic emergence worldwide (Siu et al., 2012). Usually, most hvKps have remained susceptible to various routinely used antibiotics except ampicillin. However, multidrug-resistant (MDR) hvKps have been increasingly reported globally (Zhang Y. et al., 2016; Hao et al., 2020). Additionally, as MDR and hypervirulence plasmids spread worldwide, some cKps obtained hypervirulence plasmids to create hvKp strains, whereas hvKps acquired MDR plasmids to form MDR-hvKp, and vice versa (Zhang R. et al., 2016). Recent research indicates that the spread of high-risk clonal lineages has been the primary cause of MDR-hvKp emergence, which is now recognized as a major public health issue on a global scale (Villa et al., 2017; Heiden et al., 2020). A recent study reported five cases of ST23-KL1 and ST1797-KL1 MDR-hvKps carrying carbapenemase (KPC-2) from two hospitals in China, which resulted in lethal consequences (Zhang R. et al., 2016). Because carbapenem-resistant hvKp strains may cause severe, untreatable infections in healthy individuals (Lee et al., 2017). Recently, carbapenem-resistant ST11 and ST15 hvKp have been reported from China, which caused an outbreak in the provincial hospital (He et al., 2022; Zhao et al., 2022). There have also been reports of MDR-hvKps in other parts of Asia and worldwide (Tang et al., 2020; Wyres et al., 2020; Tian et al., 2022). Because of the widespread prevalence of MDR-hvKp, it is evident that this pathogen poses a severe threat to global health.

The hvKp’s defining clinical features are multiple site infection and metastatic spread in the healthy community (Russo and Marr, 2019) and initially, a positive laboratory string test indicating a hyper-mucoid phenotype (Shon et al., 2013; Heiden et al., 2020). The definition of hypervirulence is disputable (Liu and Guo, 2019). However, a recent study identified potential plasmid-borne biomarkers, *rmpA* and *rmpA2* genes, that distinguish the two pathotypes (Russo et al., 2018). In addition, the regulator of mucoid phenotype (*rmpA*) enhances capsular polysaccharide production, resulting in hypermucoviscous phenotype and hypervirulence (Cheng et al., 2010; Choby et al., 2020). According to a recent study, changes in capsular polysaccharide production also affect some antibiotics’ susceptibilities, such as polymyxin, carbapenem, and tigecycline (Tian et al., 2022).

The hypermucoviscous phenotype of hvKp usually results from *rmpA* and *rmpA2*, located on the hypervirulence plasmid (Russo and Marr, 2019). The well-characterized virulence plasmids are pK2044, pLVPK, and Kp52.145pII (Lam et al., 2018). Although a previous study found a significant correlation between the *rmpA* gene and hypermucoviscosity, it could not clarify why some *rmpA*-positive isolates did not demonstrate the hypermucoviscous phenotype (Yu et al., 2006). However, a previous study revealed that the absence of hypermucoviscosity and reduced virulence in some *rmpA*-positive *K. pneumoniae* strains were caused by a sequential mutation of *rmpA* and *rmpA2* genes (Yu et al., 2015). Meanwhile, previous studies reported some MDR-hvKp isolates that lacked both *rmpA* and *rmpA2* biomarkers but carried others virulence factors, aerobactin and salmochelin and showed hypermucoviscous phenotype (Wozniak et al., 2019; Altayb et al., 2022).

It is undoubtedly proven that the regulator of mucoid phenotype gene (*rmpA* and *rmpA2*) along with siderophore coding genes, (aerobaction and salmochelin) altogether play a critical role in hypermucoviscosity of *K. pneumoniae* strains. But, the relationship among these genes, hypermucoviscous phenotype, and relevant virulence remained a mystery. Our study focused on different hypermucoviscous phenotype isolates that lack both *rmpA* and *rmpA2* markers as well as other defined virulence factors. In this study, we studied on 23 MDR-hvKp isolates and performed molecular typing, whole genome sequencing, comparative genomics, and phenotypic experiments to determine the genetic background of MDR-hvKp without any defined virulence genes.

## 2. Materials and methods

### 2.1. Clinical isolates and microbiological assays

The definition and screening of MDR-hvKps are still controversial (Liu and Guo, 2019). In this study, clinical investigations and several factors were considered. *K. pneumoniae* isolates that resisted at least three antibiotic groups was considered MDR-Kp (Magiorakos et al., 2012). Furthermore, MDR-Kp isolates that showed and/or any of the following phenotypes, such as hyper mucoid phenotype (carried *rmpA* and/or *rmpA2*) (Yu et al., 2006; Russo and Marr, 2019), similar growth pattern with hypervirulent isolate NTUH-K2044 (Hu et al., 2022), hypervirulence phenotype in *Galleria mellonella* infection model (Shen et al., 2019), were considered as MDR-hvKps.

Considering the above factors, 23 MDR-hvKp isolates were collected from the clinical microbiology laboratory of the first affiliated hospital of University of Science and Technology of China (USTC), Anhui, China. These critically infected patients were admitted between August 2022 and January 2023 (Supplementary Table S1). Isolates were streaked on 5% sheep agar plates and cultured at 37°C overnight to isolate the pure bacterial clones, followed by standard procedures. A VITEK 2 Compact System (bioMérieux, France) was utilized to identify the correct isolates. All isolates are stored in 40% (v/v) glycerol broth at a cryo-refrigerator (−80°C) until further experiments.

### 2.2. Antimicrobial susceptibility test

Antibiotic susceptibility of all clinical isolates was determined by previously described methods with slight modifications (He et al., 2022). The MICs of routinely used antibiotics (Supplementary Table S2), were determined by the VITEK 2 Compact System and the results of the antimicrobial susceptibility tests were interpreted according to the Clinical Laboratory Standards Institute (CLSI, 2022).

*K. pneumoniae* was cultured overnight in LB liquid medium at 37°C and 220 rpm. 0.5 μL of overnight cultured *K. pneumoniae* was streaked on LB agar plates and then incubated at 37°C for 24 h. Several monoclonal strains were selected to adjust the concentration of the bacteria in the MH (Mueller-Hinton Broth) medium (Wiegand et al., 2008). The final inoculum size for broth dilution was 5 × 10^5^ colony forming units (CFUs)/well after inoculating into MH medium with various concentrations of polymyxin B. Each concentration gradient was divided into three parallel groups and grown at 37°C and 220 rpm with shaking for 24 and 48 h. The experiment was independently repeated three times, and three technical replicates were included each time.

### 2.3. Molecular typing

Multi-locus sequence typing (MLST) and capsular K-typing of all clinical hvKp isolates were determined by previously described PCR amplification methods with some modifications (Zhao et al., 2022). First, extract the whole genome from overnight bacterial culture following the manufacturer’s protocol with minor changes (GenStar). For MLST and K typing, genomic DNA from all clinical isolates was used as templates and performed PCR using 7 pairs of housekeeping genes (*infB, phoE, pgi, tonB, mdh, gapA, rpoB*) and a pair of *wzi* primer, respectively (Table 1). The PCR conditions and parameters were followed by the manufacturer’s protocol 2 × Rapid Taq Master Mix (Vazyme). Finally, the PCR product was sequenced through sanger sequencing using gene specific primers (Table 1), by General Biotechnology, China. The sequenced outcome was analyzed by SnapGene software version 5.2. ST sequences and capsular typing are available for comparison in the database.^1^

**Table 1:** Primers used in this study.

Target gene	Primer name	Sequence (5′ to 3′)	Amplicon size (bp) approx…
*rpoB*	*rpoB* F	GGCGAAATGGCWGAGAACCA	501
	*rpoB* R	GAGTCTTCGAAGTTGTAACC	
*gapA*	*gapA* F	CAGGAAACAGCTATGACC	450
	*gapA* R	GGTAACGCCAGGGTTTTCC	
*mdh*	*mdh* F	CCCAACTCGCTTCAGGTTCAG	477
	*mdh* R	CCGTTTTTCCCCAGCAGCAG	
*pgi*	*pgi* F	GAGAAAAACCTGCCTGTACTGCTGGC	432
	*pgi* R	CGCGCCACGCTTTATAGCGGTTAAT	
*phoE*	*phoE* F	ACCTACCGCAACACCGACTTCTTCGG	420
	*phoE* R	TGATCAGAACTGGTAGGTGAT	
*infB*	*infB* F	CTCGCTGCTGGACTATATTCG	318
	*infB* R	CGCTTTCAGCTCAAGAACTTC	
*tonB*	*tonB* F	CTTTATACCTCGGTACATCAGGTT	414
	*tonB* R	ATTCGCCGGCTGRGCRGAGAG	
*wzi*	*wzi*-F	ATGATAAAAATTGCGCGCAT	447
	*wzi*-R	GCGTGATCCGTTGCTGATCC	
*rmpA*	*rmpA* F	CAAGGATGTAAACATAGTGTTG	633
	*rmpA* R	CTAAATACTTGGCATGAGCC	
*rmpA2*	*rmpA2* F	GCAATAAGGATGTTACATTAGTG	300
	*rmpA2* R	CCTTTAGGATAAAACTTCTCCC	

N.B: ‘F’ indicates forward primer and ‘R’ indicates reverse primer.

approx… indicates approximate length of amplicon size, because standard ST and K-typing genes contains several alleles.

### 2.4. Determination of hyper mucoid isolates

Regulator of mucoid phenotype bio-markers (*rmpA* and *rmpA2*) has been widely recognized till now to determine the *K. pneumoniae* hypermucoviscous phenotype. First, the whole genome was extracted from overnight bacterial culture, followed by the manufacturer’s protocol with minor modifications (GenStar). Genomic DNA from all clinical isolates was used as templates and performed PCR using two pairs of primers (*rmpA* and *rmpA2*) (Table 1). The PCR conditions and parameters were followed by the manufacturer’s protocol 2 × Rapid Taq Master Mix (Vazyme). Finally, the PCR product was run in 1% agarose gel. Isolates with target PCR band indicated hypermucoviscous phenotype positive.

### 2.5. Mucoviscosity assay

The viscosity of *K. pneumoniae* was determined using the string test. Strains that formed strings 5 mm or longer after stretching with the tip of a sterile inoculation loop were considered to have a hypermucoviscous phenotype (Shon et al., 2013). *K. pneumoniae* was cultured overnight in LB liquid medium at 37°C and 220 rpm. The cultures were diluted the following day to an OD_600_ of 1 and centrifuged at 2,350 × g for 5 min, and the OD_600_ of the supernatant was measured every minute by Gen5 Microplate Reader and Imager Software (BioTek Instruments, https://www.biotek.com/). Experiments were performed using three technical replicates and three biological replicates.

### 2.6. *Galleria mellonella* infection model

The virulence of *K. pneumoniae* isolates was evaluated using the *Galleria mellonella* infection model (Shen et al., 2019). Larvae (0.3–0.4 g) were stored in the dark and used within 3 days after shipment (Tianjin Huiyude Biotechnology Co., Ltd.). Before injection, the bacterial pellet was washed with sterile saline and diluted to 1 × 10^8^ CFU/mL. Using a 1 mL insulin syringe, 10 μL of the bacterial suspension was injected into the center of the second gastropod of the larvae. A group of 10 larvae was randomly selected for injection. A group of larvae was injected with 0.9% NaCl solution as the negative control. Another group of larvae without injection was also included parallel. After injection, the larvae were incubated at room temperature, and survival rate was monitored daily up to 72 h. Death was recorded when the larvae no longer responded to touch. In all cases, no dead larvae were observed in the negative control groups. This experiment was repeated three times independently.

### 2.7. Growth curves

The growth curves of *K. pneumoniae* were established in LB medium manually, followed by standard protocol with minor modifications. Overnight cultures were diluted to an OD_600_ of 0.02 and grown in 96-well plates at 37°C and 220 rpm with shaking. The absorbance of the culture solution at OD_600_ was measured every 0.5 h until it reached the peak and became flat. The OD_600_ was measured by Gen5 Microplate Reader and Imager Software (BioTek Instruments, https://www.biotek.com/). Experiments were performed using three technical replicates and three biological replicates.

### 2.8. Whole genome sequencing, assembly, and annotation

A total of 23 *K. pneumoniae* isolates were sequenced by second-generation technology. Briefly, whole genome sequencing of *K. pneumoniae* was performed using an Illumina Hi-Seq 4,000 platform at Nuosai Jiyin Zu Research Center Limited Company (Beijing, China). The fastp is used to filter the raw sequencing data (Chen et al., 2018). Filtered sequence data were assembled using Unicycler v0.4.8 (Wick et al., 2017) and annotated with the rapid prokaryotic genome annotation tool, Prokka 1.14.6 (Seemann, 2014).

### 2.9. Genome profiling and comparative genomics analysis

ABRicate version 1.0.14 was implemented to detect acquired antimicrobial resistance genes (ARGs) by aligning genomic sequences to the ResFinder and NCBI databases (Zankari et al., 2012). Kleborate and ABRicate were utilized to determine the isolates’ virulence factors by aligning their genomic sequences to the VFDB database (Zankari et al., 2012; Wyres et al., 2016). Multi-locus sequence typing (MLST) was conducted by MLST 2.15 (Jolley and Maiden, 2010). Capsule typing was undertaken by Kleborate (Wyres et al., 2016; Lam et al., 2021). The HarvestTools kit (Parsnp, Gingr, and HarvestTools) and BacWGSTdb were utilized to perform a comparative genomic and phylogenetic analysis of the different isolates to build phylogenetic trees based on the maximum likelihood method (Treangen et al., 2014; Feng et al., 2021). The sequence alignment of mutant *cps* gene cluster was visualized using Easyfig 2.2.5 (Sullivan et al., 2011). The interactive tree of life (iTOL) v5 was used to construct a phylogenetic tree (Letunic and Bork, 2021). SNPs among various strains were investigated using Snippy.^2^ Heatmaps were illustrated with the ComplexHeatmap R package (Gu et al., 2016).

### 2.10. Genome accession number

Whole genome sequencing data were deposited in the NCBI database under BioProject: PRJNA971333.

### 2.11. Statistical analysis

All the statistical analyses were performed using GraphPad Prism 7.0.^3^ Error bars represent SEM. All the experiments were performed at least three times with three technical replicates.

## 3. Results

### 3.1. Clinical and molecular insights of MDR-hvKp isolates

A total of 23 MDR-hvKp isolates were collected. Most of the patients’ age is above 60 years (*n* = 16), ≥40- ≤ 60 (*n* = 4) and young individuals (*n* = 3), where 78.3% were male (*n* = 18), and 21.7% were female (*n* = 5). Interestingly, in clinical investigation, the majority of patients were hospitalized for other than lung diseases or its associated pneumonia (*n* = 7) and most of them were hospitalized due to old age diseases or others (*n* = 16) that indicates nosocomial infection (Supplementary Table S1). Clinically, most of the isolates were collected from sputum (*n* = 20) and remaining from blood (*n* = 3). These 3 patients were admitted in ICU for rather than *K. pneumoniae* associated diseases. In antibiotic susceptibility test, all isolates showed resistance to Cefuroxime axetil, Cefuroxime, Ceftriaxone, and intermediate resistance to Polymyxin B (Supplementary Table S2). In multi-locus sequence typing (MLST) and capsular typing (K-typing), nine isolates belonged to ST11 and all were KL64 except one belonged to KL47, five to ST23 (KL1), two to ST307 (KL102), and remaining belonged to an individual ST e,g. ST15 (KL19), ST 86 (KL2), ST 147 (KL64), ST485 (NA) and ST3132 (KL24) (Supplementary Table S3). In addition, the WGS results indicated that one isolate belonged to *Klebsiella quasipneumoniae* (22100407).

### 3.2. Isolates 22122315 (ST15) and 22091569 (ST307) lacked both *rmpA* and *rmpA2* but still demonstrate hypermucoviscosity and hypervirulence

**Figure 1:**
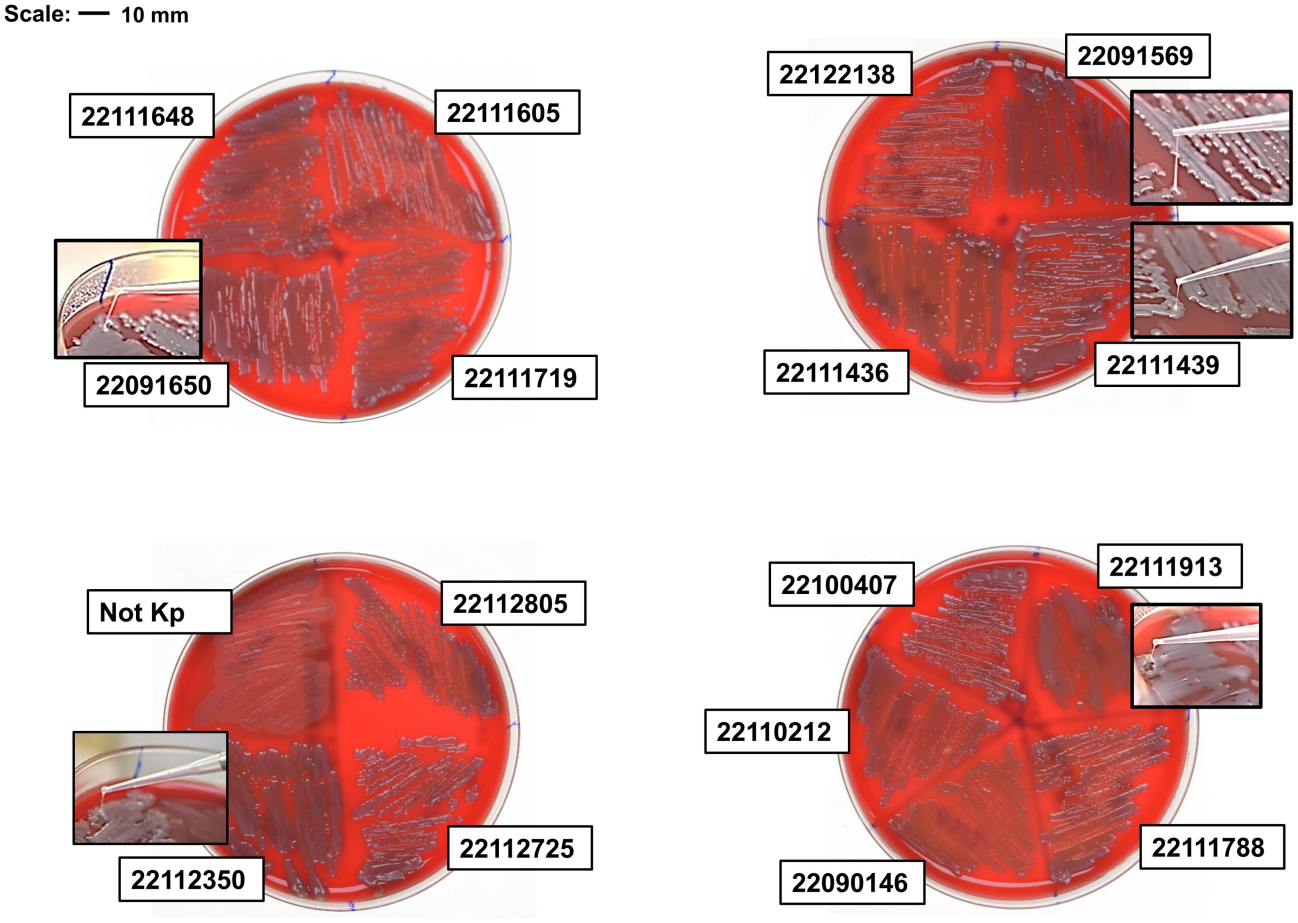
String test of clinical isolates on blood agar plate. Hypervirulent isolate 22091569 formed the highest length of string. Besides, isolate 22091650, 22112350, 22111436 and 22111913 also formed string.

The presence of regulator of mucoid phenotype A (*rmpA*) and A2 (*rmpA2*) positively determines hyper-mucoid phenotype and hypermucoviscosity. Of 23 isolates, nine isolates showed *rmpA* positive and 14 isolates showed *rmpA2* positive where nine isolates showed both *rmpA* and *rmpA2* positive, and 7 isolates showed negative to both (Supplementary Figures S1, S2). Isolates with both genes showed hyper-mucoid phenotype and mucoviscosity in centrifuge at 2350 × g (Supplementary Figure S3). Interestingly, two isolates, 22122315 (ST15) and 22091569 (ST307), that lacked both genes showed hyper-mucoid phenotype. Meanwhile, isolate 22091569 showed the highest string length in the blood agar plate after overnight incubation at 37°C, whereas isolate 22122315 showed stickiness, but the string length was not significant (≤ 5 mm) (Figure 1).

We then investigated some phenotypic experiments, e.g., mucoviscosity assay, growth curve, and including *G. mellonela* infection model to assess its virulence. NTUH-K2044 was used as a reference hypervirulent isolate and positive control.

**Figure 2:**
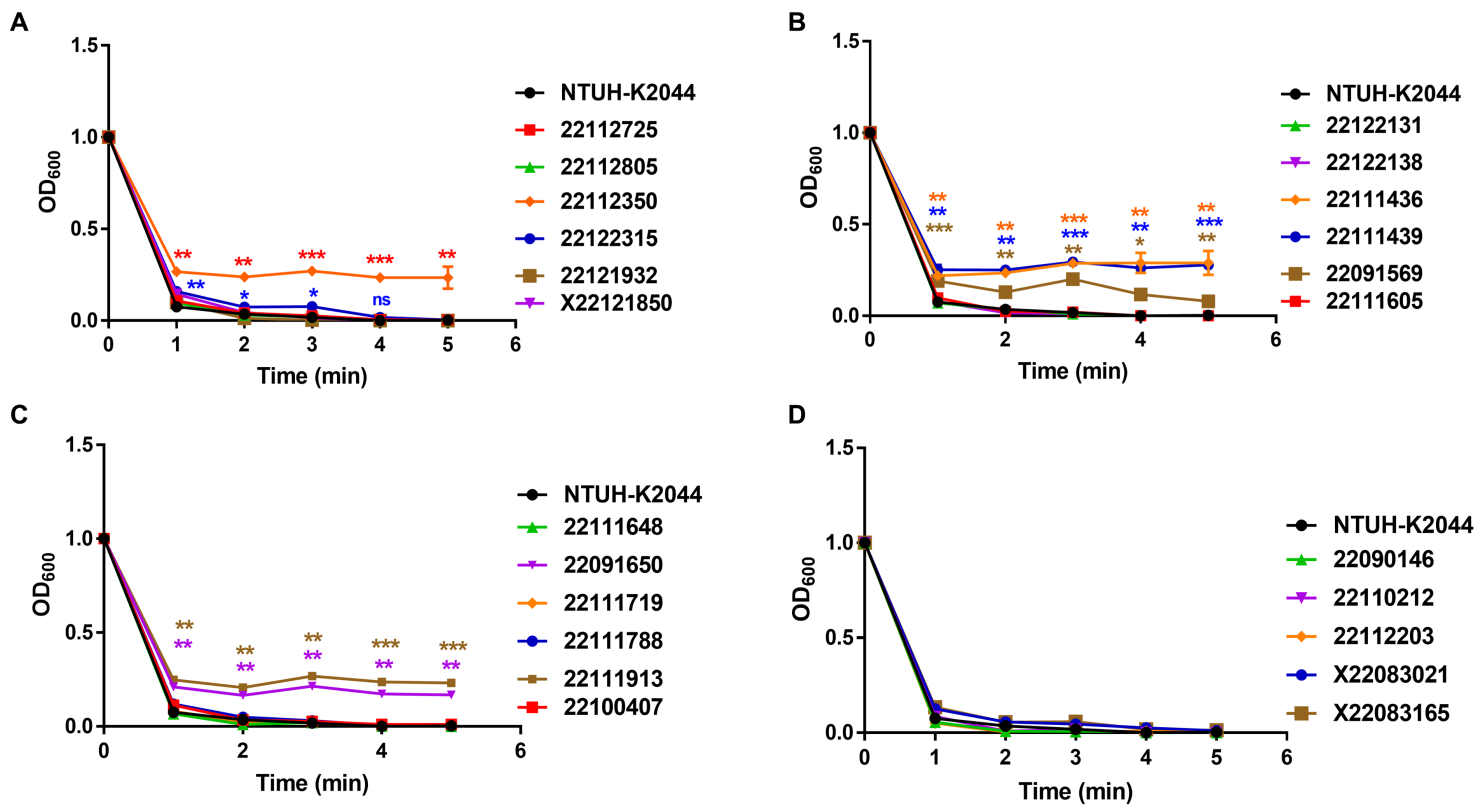
Mucoviscosity assay of MDR-hvKp clinical isolates **(A–D)**. Viscosity levels were determined by the OD_600_ of the supernatant obtained after centrifugation of the overnight culture at 2,350 × g for 5 min. Mucoviscosity was compared with classical hypervirulent isolate NTUH-K2044. *, *p* < 0.05, **, *p* < 0.01, ***, *p* < 0.001, ****, *p* < 0.0001, ns, not significant.

In the mucoviscosity assay, all the isolates, including NTUH-K2044, were incubated overnight, adjusted OD (OD_600_ = 1) and centrifuged at low speed (2,350 × g) for 5 min, where measured the OD_600_ after every single minute. The supernatant of NTUH-K2044 became absolutely transparent after 3 min. Alternatively, isolate 22112350 remained turbid, and no pellet was formed after 5 min, whereas isolate 22122315 showed a significant mucoviscosity up to 3 min but still exhibited a mucoid pellet (Figure 2A and Supplementary Figure S3A). In addition, 22111436 and 22111439, and 22091569 showed hypermucoviscosity and remained turbid; no pellet was formed after 5 min (Figure 2B and Supplementary Figure S3B). In isolates 22091650 and 22111913, it remained hyper-mucoid and showed hypermucoviscosity compared to the control (Figure 2C and Supplementary Figures S3B,C). Furthermore, isolates X22083021 and X22083165 showed slight stickiness compared to the control (Figure 2D and Supplementary Figure S3C). Here, except isolates 22122315 and 22091569, all the hyper-mucoid isolates had both *rmpA* and *rmpA2* gene.

**Figure 3:**
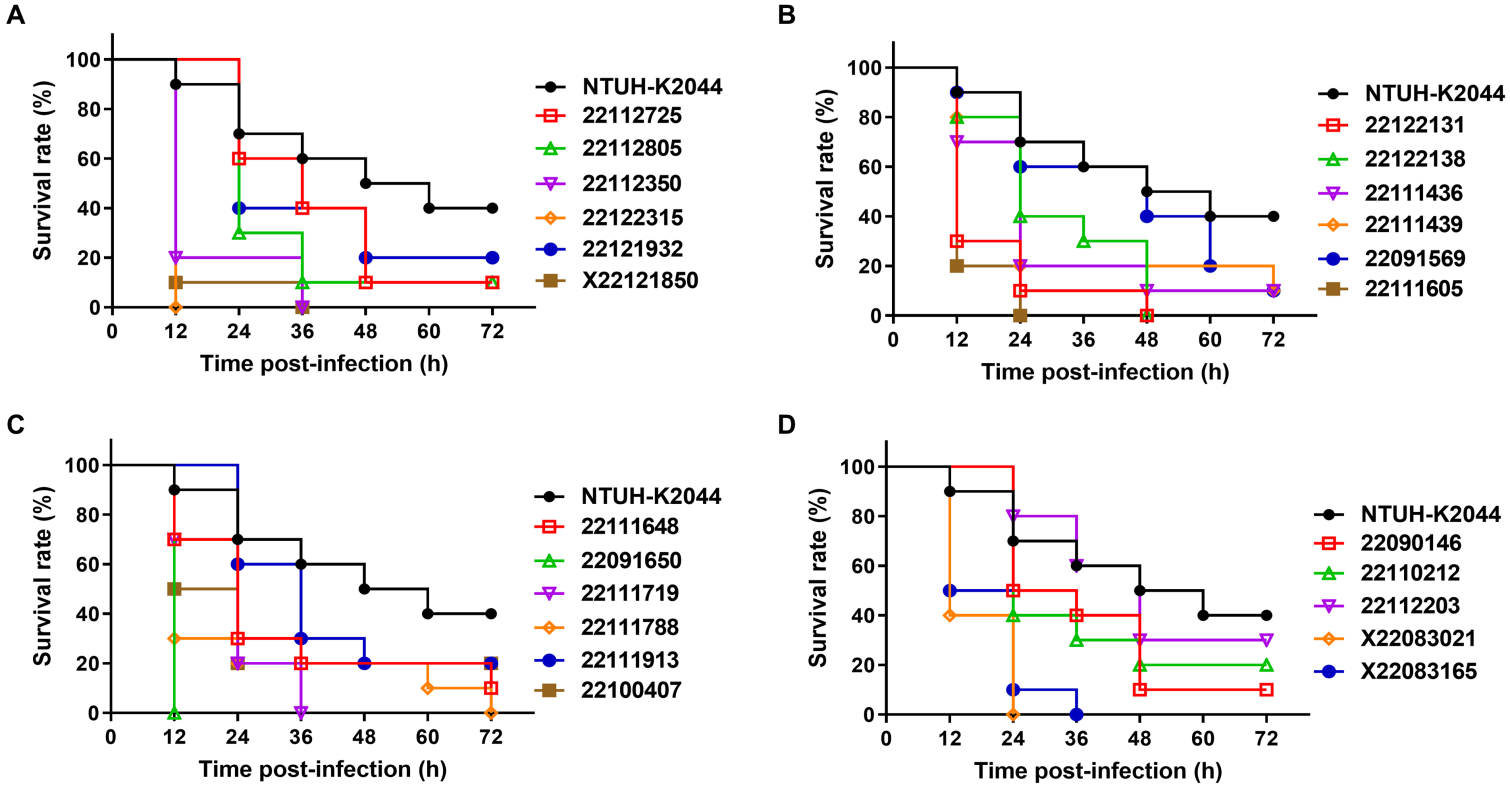
Virulence assay of MDR-hvKp isolates in the *Galleria mellonella* infection model **(A–D)**. Survival rate of wax moth was calculated after 72 h post infection by the injection of MDR-hvKp isolates (1 × 10^6^ CFU). Virulence was assessed compared to NTUH-K2044 (control).

In *G. mellonella* infection model, all the clinical MDR-hvKp isolates demonstrated more virulence compared to NTUH-K2044 where 40% (±10) of insects survived after 72 h post-infection (Figure 3 and Supplementary Table S4). Specifically, isolates 22122315 (and 22091650) showed the highest virulence and all larvae were died after 12 h post-infection (mean survival rate, 0% (±0)). Meanwhile, isolate 22091569 showed higher virulence compared to control, where only 10% (±10) of injected larvae remained alive after 72 h post-infection. Moreover, for isolates 22111605 and X22083021, all the larvae died after 24 h post-infection. All the larvae were died after 36 h post-infection in case of isolates, X22121850, 22112350, 22111719, and X22083165. This infection model indicated both *rmpA*/ *rmpA2* lacked isolates 22122315 and 22091569 showed hypervirulence compared to control (Supplementary Table S4).

In growth curve, NTUH-K2044 showed the fastest growth rate, and all the clinical isolates showed similar growth patterns except isolate X22083021, which reached the peak at 8.5 h and declined at 9 h and then remained stable (Supplementary Figure S4).

### 3.3. Comparative genomic analysis reveals hypervirulent isolates, 22122315 and 22091569 lacked virulence factors

**Figure 4:**
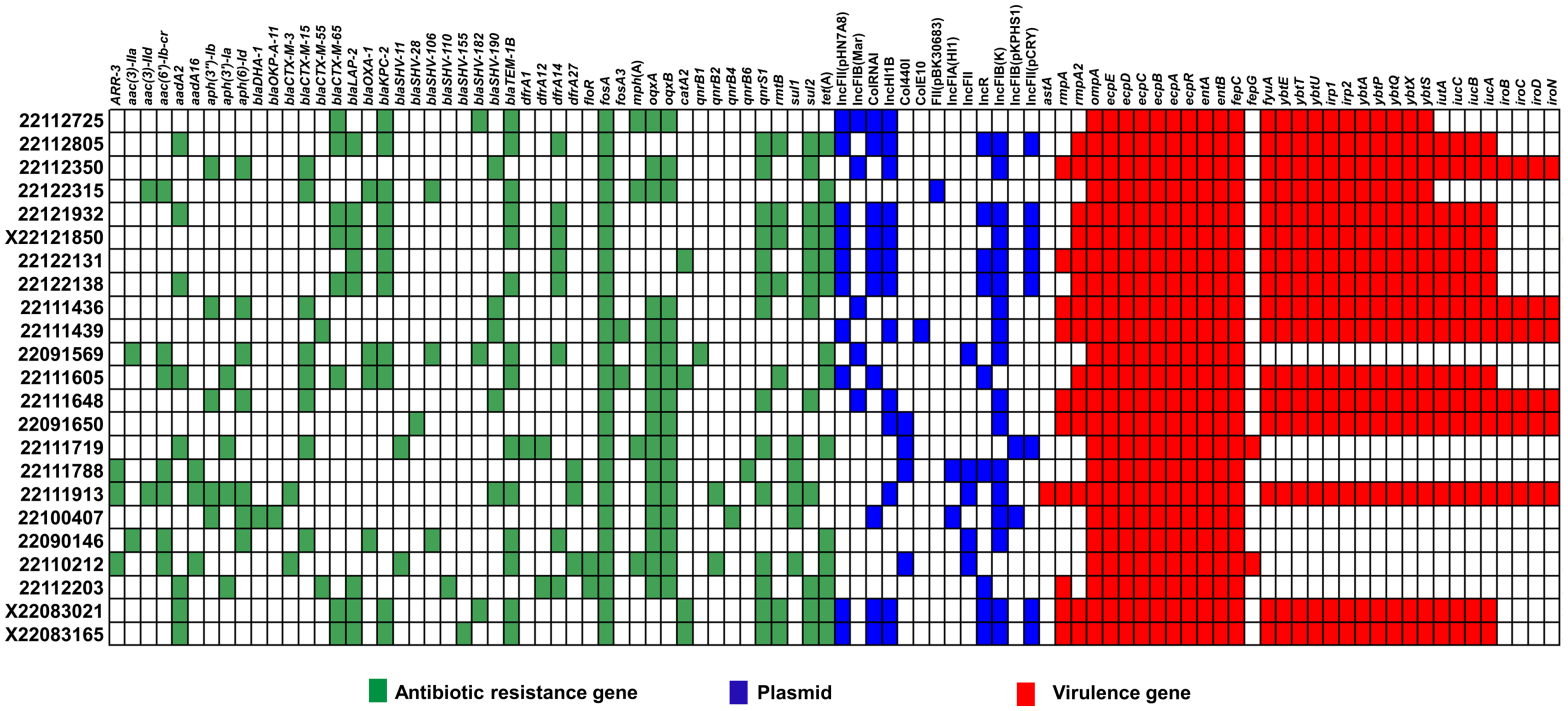
Comparative genomics of clinical MDR-hvKp isolates. Here, antimicrobial resistance genes (blue), virulence factors (red) and plasmid types (blue) are represented in different colors.

We then performed WGS to explore functional genomics and better understanding the reasons behind the hyper-mucoid phenotype and mucoviscosity. In comparative genomics, we analyzed the distribution of antibiotic resistance genes, plasmid, and virulence factors among the isolates (see Figure 4).

We identified 46 MDR genes across the MDR-hvKp clinical isolates, 17 of which encoded β-lactamases, including carbapenemase-encoding genes. Moreover, the MDR-hvKp isolates carried β-lactamase-encoding genes *bla*_TEM − 1B_ (*n* = 14), *bla*_LAP − 2_ (*n* = 8) and *bla*_OXA − 1_ (*n* = 5), ESBL encoding genes, *bla*_CTX − M − 15_ (*n* = 8), *bla*_CTX − M − 65_ (*n* = 8), *bla*_SHV − 190_ (*n* = 5) and carbapenemase-encoding genes *bla*_KPC − 2_ (*n* = 11). In addition, we noticed all the MDR-hvKp isolates carried the fosfomycin resistance gene, *fosA*.

Meanwhile, both isolates 22122315 and 22091569 showed MDR phenotype in antibiotic susceptibility testing and the comparative genomic analysis of antibiotic resistant genes also confirmed this phenomenon. Both isolates carried MDR genes such as β-lactamase-encoding genes *bla*_TEM − 1B_, and *bla*_OXA − 1_, carbapenemase-encoding genes, *bla*_KPC − 2_, ESBL encoding genes, *bla*_CTX − M − 15_, *bla*_SHV − 106_, quinolone efflux pump coding genes, *oqxAB*, tetracycline resistance genes, *tet*(A) (Figure 4). However, none of these isolates carried any polymyxin-resistant gene, *mcr*, and antibiotic susceptibility test also confirmed it.

Furthermore, majority of isolates carried IncFIB(K) plasmid. In addition, some hypervirulent isolates also carried IncFII(pHN7A8), IncFIB(Mar), ColRNAI, and IncHI1B plasmids. Meanwhile, isolate 22091569 carried IncFIB(Mar), IncFII, and IncFIB(K) plasmid, whereas 22122315 carried FII(pBK30683) only.

In case of virulence factors, all the MDR-hvKp isolates carried outer membrane protein-coding genes (*ompA*), fimbriae encoding genes (*ecpABCDER*), enterobactin coding genes (*entAB*, and *fepC*). Besides, the majority of isolates carried yersiniabactin coding genes (*fyuA, ybtE, ybtT, ybtU, irp1, irp2, ybtA, ybtP, ybtQ, ybtX, ybtS*) (69.5%). In addition, aerobactin coding genes (*iutA*, *iucABC*), salmochelin coding genes *(iroBCD*, and *iroN*) were also present in a few isolates. However, the virulence-defined genes were not prevalent in all phenotypically observed hypervirulence isolates in *G. mellonella* infection models. We found that isolates 22122315 and 22091569 lacked virulence-defined genes, aerobactin and salmochelin. However, the 22122315 isolates alone had yersiniabactin coding genes.

### 3.4. Single nucleotide polymorphism (SNP) analysis indicates both two isolates carried a novel mutation in *wzc* of the *cps* gene cluster

Among the 23 MDR-hvKp isolates, 22122315 and 22091569 showed hypervirulence and hyper-mucoid phenotype without regulators of mucoid phenotype gene, *rmpA* and *rmpA2*. Usually, these two biomarkers are positively responsible for hypermucoviscosity in *K. pneumoniae*. We performed single nucleotide polymorphism (SNP) analysis using WGS to reveal its mystery.

**Figure 5:**
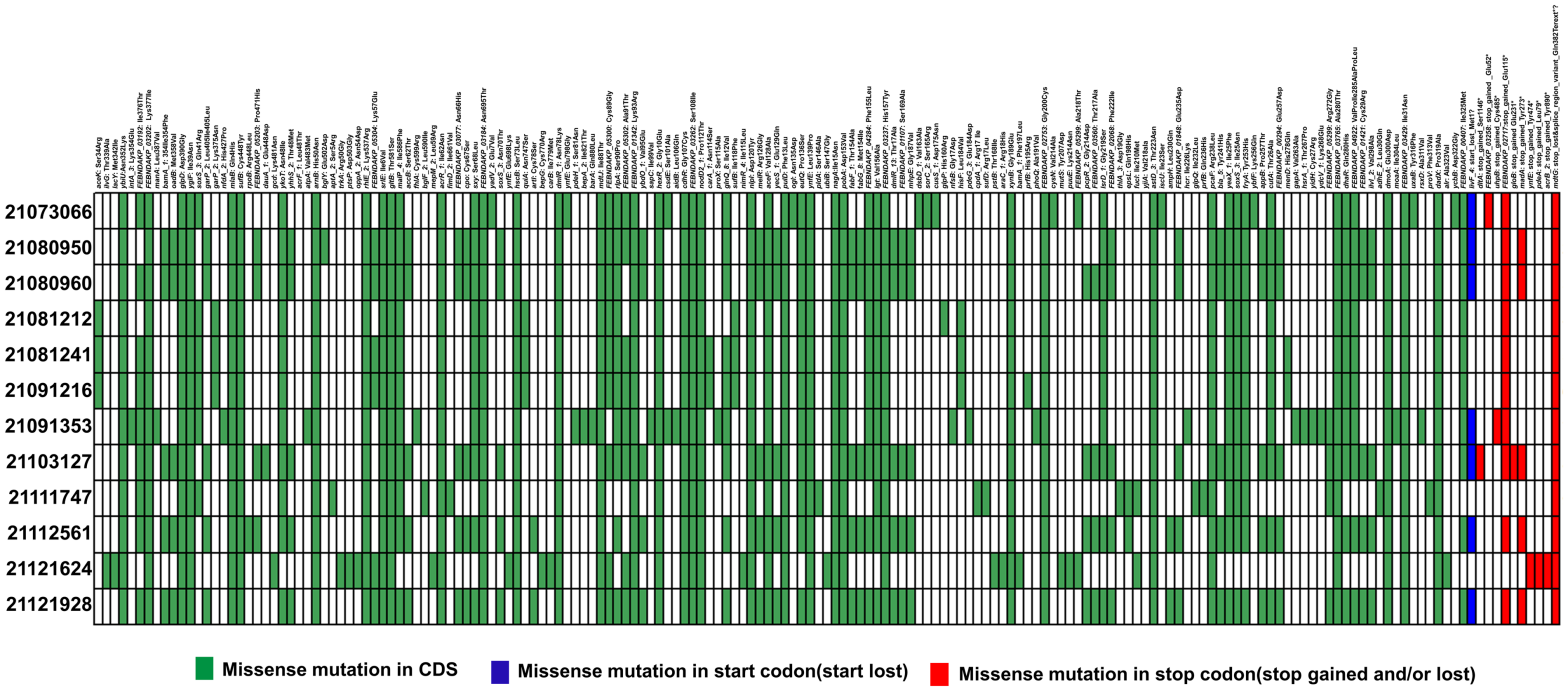
Distribution of single nucleotide polymorphisms (SNPs) in hypervirulent isolate 22122315 that causes missense mutations compared to previous ST15 isolates (Zhao et al. 2022). Here, presence or absence of missense mutations in CDS region (green), start codon (blue) and stop codon (red) are represented in different colors.

When analyzed SNPs in 22122315 (ST15), it was compared with recently reported ST15 isolates (Zhao et al., 2022). Considering all SNPs in the coding DNA sequence (CDS) region, we identified 36 common mutations, including 1 stop lost. Moreover, we identified 8 missense novel mutations in unidentified locus among the 36 repetitive missense mutations, and all the unknown mutant genes produce hypothetical proteins except *FEBNDAKP_03184* (Supplementary Table S5). In 2084th nucleotide position, adenine(A) mutated to cytosine(C), resulting in a change of 695th amino acid from Asparagine (Asn) to Threonine (Thr) (Figure 5). Interestingly, this mutant gene is responsible for putative tyrosine-protein kinase in the *cps* region.

**Figure 6:**
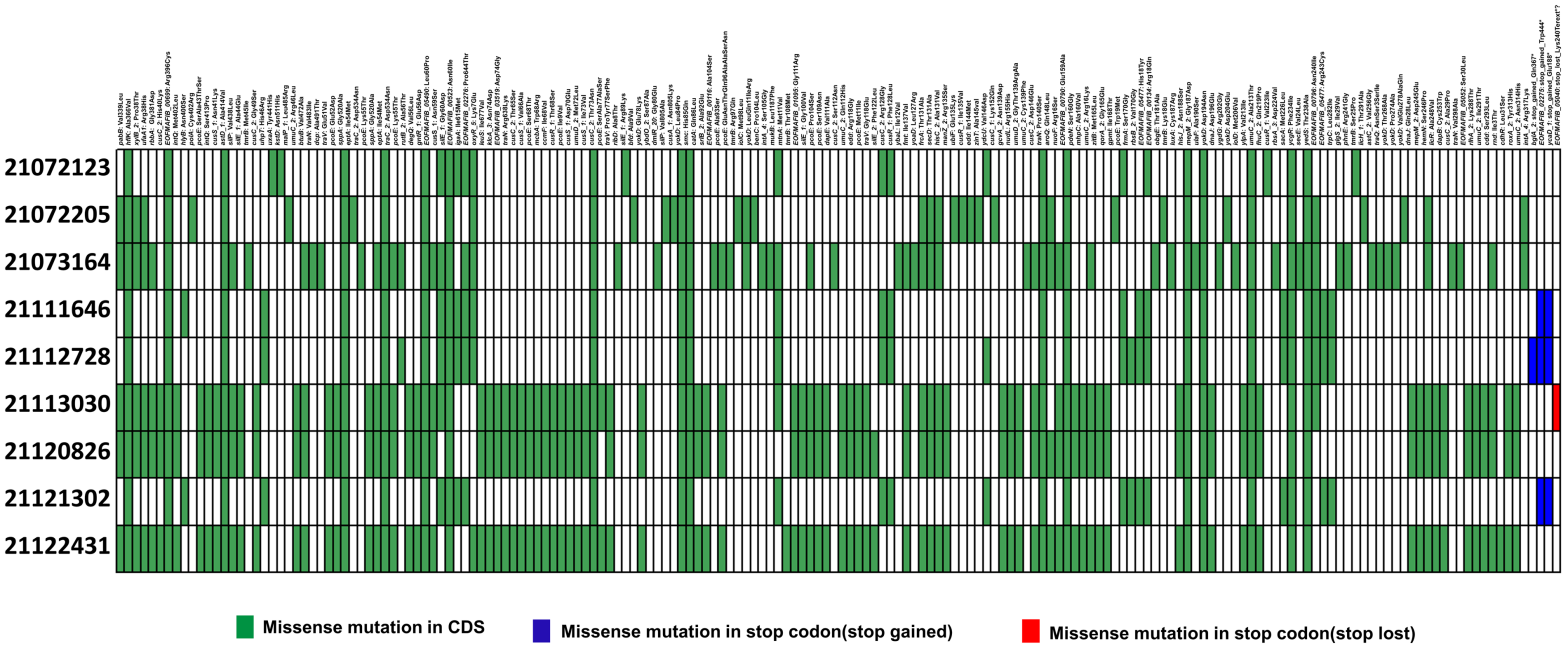
Distribution of single nucleotide polymorphisms (SNPs) in hypervirulent isolate isolate 22091569 compared to previous ST307 isolates (He et al., 2022). Here, presence or absence of missense mutations in CDS region (green), start codon (blue) and stop codon (red) are represented in different colors.

Similarly, to detect SNPs in 22091569, it was compared with ST307 isolates (He et al., 2022). Here, we identified 17 mutations were same in each ST307 isolates. Among them, 4 mutations were found in unknown locus, namely, *EOFMAFIB_00699* (1186C > T, Arg396Cys), *EOFMAFIB_00523* (179A > T, Asn60Ile), *EOFMAFIB_00116* (310G > T, Ala104Ser), and *EOFMAFIB_00796* (c. 719A>T. p. Asn240Ile) that produce a hypothetical protein (Supplementary Table S6). Apart from this, we identified a novel mutation in *EOFMAFIB_02276* loci where the 1930th nucleotide was mutated from cytosine (C) to adenine (A) which resulted in a change of 644th amino acid Proline (Pro) to Threonine (Thr) (Figure 6). Interestingly, this mutation is also responsible for putative tyrosine-protein kinase in the *cps* region, the same as in isolate 22122315.

**Figure 7:**
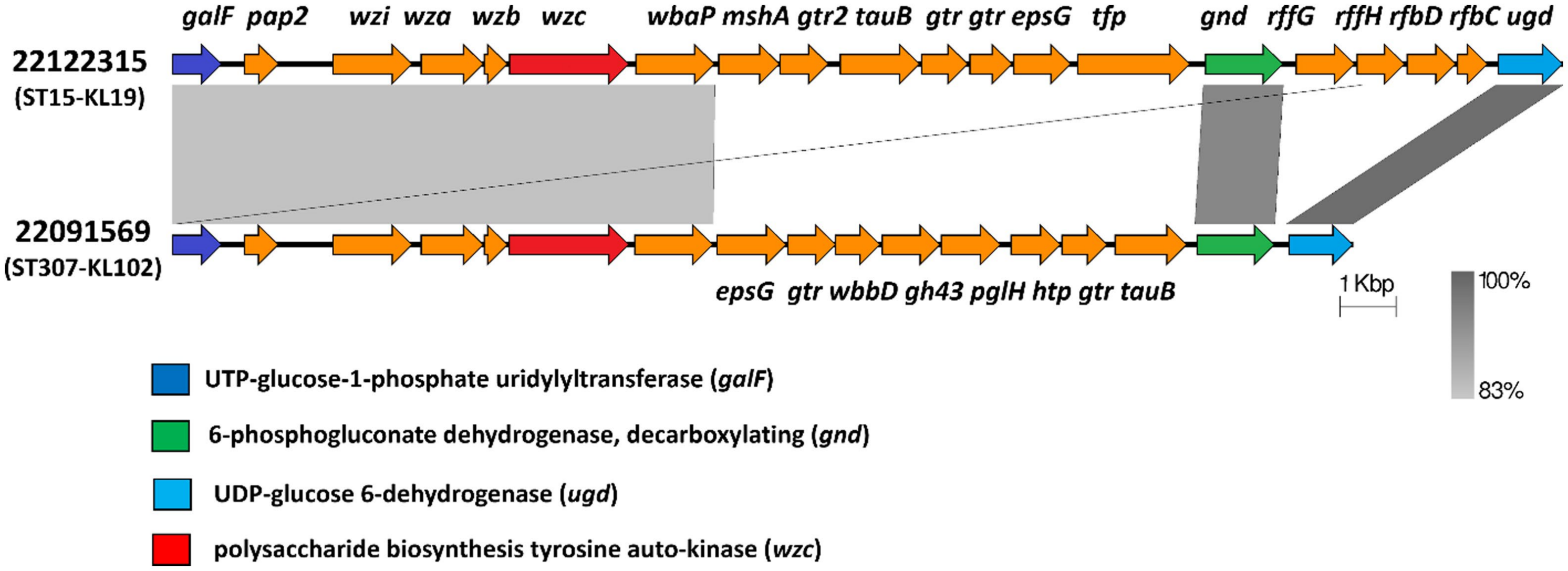
Mutant *wzc* of the *cps* gene cluster in both isolates 22122315 and 22091569. Both isolates carried a novel mutation in *wzc* (red) of *cps* region that extends from *galF* (dark blue) to *gnd* (green) and to *ugd* (light blue). (*galF*-UTP--glucose-1-phosphate uridylyltransferase, *pap*2- phosphatase family protein, *wzi*- capsule assembly family protein, *wza*- polysaccharide export protein, *wzb*- low molecular weightprotein-tyrosine-phosphatase, *wzc*-polysaccharide biosynthesis tyrosine autokinase, *wbaP*- undecaprenyl-phosphate galactose phosphotransferase, *msh*A- D-inositol-3-phosphate glycosyltransferase, *gtr*2- glycosyltransferase family 2 protein, *tua*B- Teichuronic acid biosynthesis protein, *gtr*- glycosyl transferase, *epsG*-EpsG family protein, tfp-tail fiber protein, *wbbD*- UDP-Gal: alpha-D-GlcNAc-diphosphoundecaprenol beta-1,3-galactosyltransferase, *gh4*3-GH43glycoside hydrolase family 43 protein, *pglH*- GalNAc-alpha-(1->4)-GalNAc-alpha-(1->3)-diNAcBac-PP-undecaprenol alpha-1,4-*N*-acetyl-D-galactosaminyltransferase, htp-hypothetical protein, *tuaB*- Teichuronic acid biosynthesis protein TuaB, *gnd*-6-phosphogluconate dehydrogenase,decarboxylating, *rffG*- dTDP-glucose 4,6-dehydratase 2, *rffH*- Glucose-1-phosphate thymidylyltransferase 2, *rfbD*- dTDP-4-dehydrorhamnose reductase, *rfbC*-dTDP-4-dehydrorhamnose 3,5-epimerase, *ugd*- UDP-glucose 6-dehydrogenase).

Furthermore, we examined the mutation of both loci, *FEBNDAKP_03184* and *EOFMAFIB_02276*, in the *cps* gene cluster. This cluster ranges from *galF* (UTP--glucose-1-phosphate uridylyltransferase) to *ugd* (UDP-glucose dehydrogenase). Isolate 22122315 contained a set of 20 genes within the *cps* gene cluster while 22091569 had 17 genes. NCBI blast analysis showed that both mutant loci carried 720 amino acids and were located in the *wzc* of the *cps* gene cluster. Besides, *wzc* is also responsible for the tyrosine auto-kinase involved in polysaccharide biosynthesis (Figure 7).

### 3.5. Phylogenetic analysis

**Figure 8:**
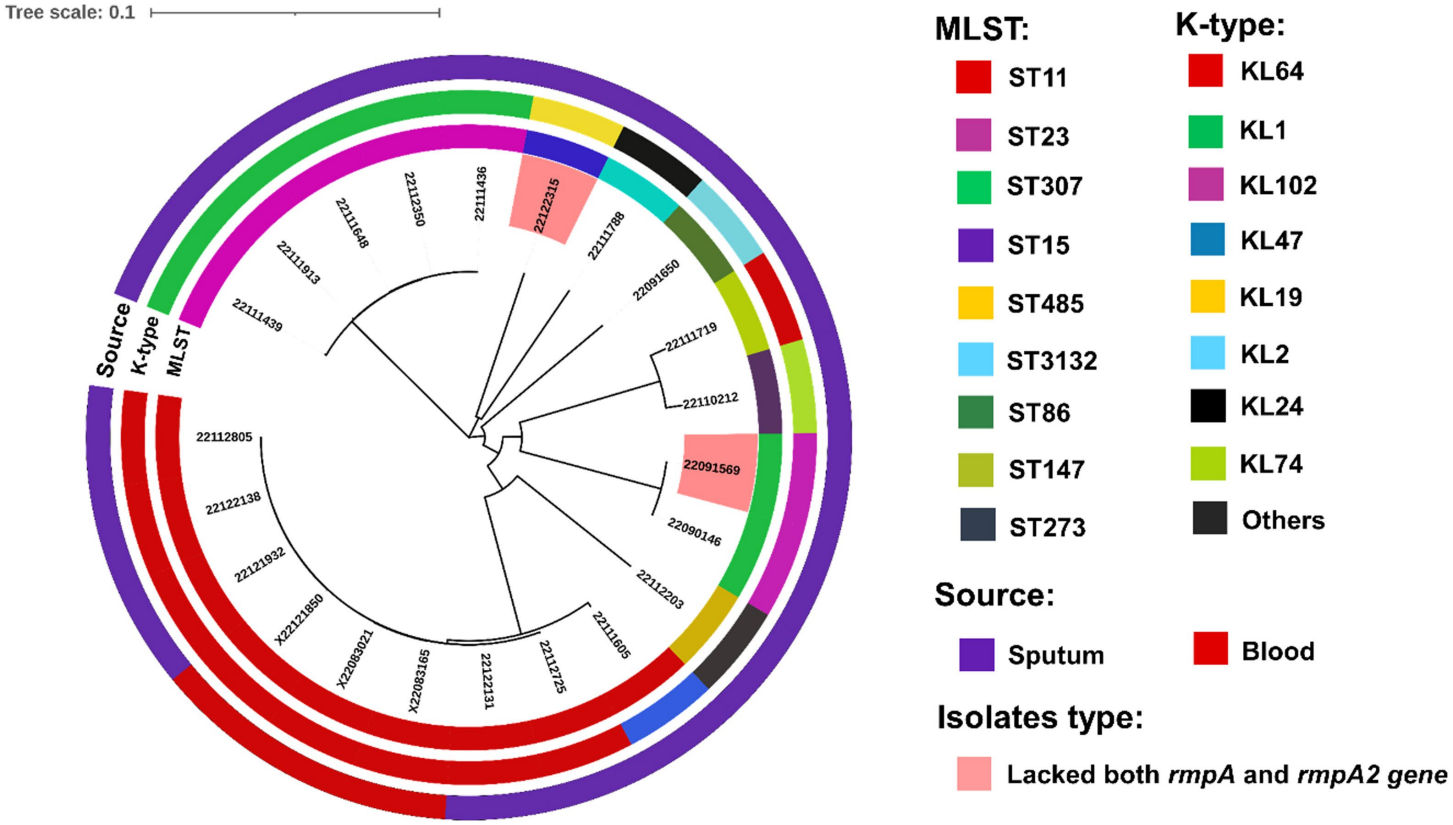
Phylogenetic analysis of clinical isolates based on the maximum likelihood approach. Isolates with different MLST and capsule types (K- type) and sources are illustrated in different colors using iTOL v5 (Letunic and Bork, 2021). Two hypervirulent isolates, 22122315 and 22091569, that lacked both *rmpA* and *rmpA2* genes and virulence factors, were also marked with a different color.

We conducted molecular phylogenetics to construct a phylogenetic tree based on the maximum likelihood approach. Moreover, the trees were evolutionary illustrated into four subgroups based on highly similar homology where, two hypervirulent isolates, 22122315 and 22091569 were originated from different sub-groups (Figure 8). The tree showed that all the ST11, ST23, ST307 isolates showed similar homology and were positioned in the same sub-groups. Interestingly, isolates 22111719 and 22110212 showed similar homology, but their ST and K-typing were not identical; they belonged to ST147 and ST273, respectively.

## 4. Discussion

It is scientifically believed that the regulator of mucoid phenotype biomarkers (*rmpA* and *rmpA2*) regulate and enhance the capsular polysaccharide synthesis, which is the main reason behind the hypervirulence and hyper-mucoid phenotype in *K. pneumoniae*. However, the emergence of hypervirulent isolates that lacked both these biomarkers challenged this common scientific belief. A previous study reported that these two plasmid-borne biomarkers were crucial in hypermucoviscosity (Russo et al., 2018). Another study reported that hvKp infections are an emerging global threat, and two plasmid-borne biomarkers positively differentiate the hypervirulence isolates from cKp (Spadar et al., 2023).

Hence, this study collected 23 MDR-hvKp isolates and performed molecular epidemiological analysis, phenotypic experiments, and *the G. mellonella* infection model to reveal its genetic and phenotypic insights. Clinically, most of the patients were above 60 years, male, and hospitalized for rather than chronic lung diseases, suggesting that immunocompromised and nosocomial infection are still the leading cause of infection (Liu et al., 2022). In molecular typing, most of the isolates belonged to ST11 (KL64) and ST23 (KL1). Previous studies also reported a ST11 outbreak of MDR-hvKp isolates from the same province (He et al., 2022), and moreover, a ST23 outbreak has been reported in China since 2015 (Zhang R. et al., 2016). Then, we compared the *rmpA* and *rmpA2* biomarkers in phenotypically hyper-mucoid isolates. This study discovered two MDR-hvKp isolates, namely, 22112315 (ST15) and 22091569 (ST307), that showed hypervirulence and hyper-mucoid phenotype without both *rmpA* and *rmpA2* gene in their virulence plasmid. In AST, both isolates showed resistance to most routinely used antibiotics and intermediate to polymyxin B. Moreover, Polymyxin is the last line of defense against gram-negative bacteria (Li et al., 2006). This phenomenon also confronted the existing belief of MDR-hvKp; the higher the virulence, the lower the resistance (He et al., 2022).

In phenotypic experiments, isolate 22122315 showed the highest virulence in the *G. mellonella* infection model and significant mucoviscosity. Conversely, isolate 22091569 showed the highest hypermucoviscosity in the string test and higher lethality in infection model than NTUH-K2044. However, 22112315 could not form strings above 5 mm in the string test. In our study, it was observed that while some strains did not demonstrate a substantial mucoviscosity in comparison to NUTH-K2044, all MDR-hvKp strains exhibited higher virulence than NUTH-K2044 when evaluated using the *G. mellonella* infection model. Our findings differ slightly from prior studies that have reported a favorable correlation between hypermucoviscosity and hypervirulence, except for a few cases (Walker and Miller, 2020; Zhu et al., 2021).

Some previous studies also reported that the string test and *G. mellonella* infection model cannot precisely detect hvKp and suggested multiple methods (Russo et al., 2018; Russo and MacDonald, 2020). Indeed, some string test-positive isolates were previously shown to be cKP, while string test negative *K. pneumoniae* strains were hvKp (Li et al., 2019). In growth curve, all the clinical MDR-hvKp isolates showed hypervirulent growth patterns similar to NTUH-K2044 (Hu et al., 2022).

Mainly, the emergence of most MDR-hvKp is due to the strains acquiring MDR genes and hypervirulence genes (He et al., 2022). Comparative genomics showed that isolates, 22122315 and 22091569 carried carbapenemase-encoding genes *bla*_KPC − 2_, β-lactamase encoding genes, *bla*_OXA − 1_, and *bla*_TEM − 1B_, ESBL encoding genes, *bla*_CTX − M − 15_, *bla*_SHV − 106_. A recent study reported that the main driver of carbapenem resistance in *K. pneumoniae* is the acquisition of genes that encode carbapenemases such as KPC, NDM, some variants of OXA, and others (Spadar et al., 2023).

We found that isolates 22122315 and 22091569 both carried outer membrane protein-coding genes, *ompA,* fimbriae encoding genes, *ecpABCDER* and enterobactin coding genes, *entAB*, *fepC*, but lacked virulence-defined, aerobactin and salmochelin coding genes. A recent study reported a rare ST14 MDR-hvKp that lacked both hyper-mucoid regulators (*rmpA*/ *rmpA2*) but showed hypermucoviscous phenotype with the presence of *iroE* and *iroN* (salmochelin), and *iutA* (aerobactin) genes (Altayb et al., 2022). Another recent study from India also claimed a clinical isolate which exhibited hypermucoviscosity without known hypermucoviscosity deteminants (*rmpA* and *rmpD*) (Dey et al., 2022), but skipped the role of siderophore (aerobactin and salmochelin) coding genes. Another recent study found aerobactin as a promising biomarker to identify MDR-hvKp in case of a negative string test due to unstable *rmpA2* (Shankar et al., 2021). Similarly, hypervirulence is usually associated with aerobactin (*iuc*) and salmochelin (*iro*) genes carried on plasmids. The *iro* and *iuc* loci are recurrently accompanied by additional genes (e.g., *rmpA*, *rmpA2*, and *rmpC*) linked with a hypermucoviscous capsule phenotype (Russo et al., 2018; Spadar et al., 2023). Our current study discovered two isolates without any hypervirulence determinants. However, isolates without any virulence-defined genes and how they showed phenotypically hypervirulence remained a mystery, and it may be an alarming threat to global health.

Finally, SNP analysis revealed that both isolates 22122315 and 22091569 had a novel mutation in loci *FEBNDAKP_03184* (c. 2084A>C, p. Asn695Thr) and *EOFMAFIB_02276* (c. 1930C>A, p. Pro644Thr) respectively. Interestingly, the NCBI blast result indicates that both mutations are located in the *wzc* of the *cps* gene cluster that is responsible for putative tyrosine-protein kinase. Some recent studies reported that, among the capsular type defined gene in the *cps* biosynthesis gene cluster, *wzc* genes are involved in tyrosine-protein kinase (Patro et al., 2020; Yang et al., 2021). Moreover, a previous study reported that *rmpA* and *rmpA2* regulate the *cps* gene and enhance capsular polysaccharide synthesis (Choby et al., 2020). Another study reported that *wzc* is involved in the synthesis and assembly of capsular polysaccharide, and the hyperviscous phenotype of hvKp is primarily due to its overproduction (He et al., 2022). However, the current study found a mutant *cps* gene cluster in hypervirulent isolates that lacked both mucoid regulators (*rmpA*/*rmpA2*). It is scientifically established that *rmpA*/*rmpA2* regulates *cps* gene cluster and enhances capsular polysaccharide synthesis. In contrast, our current study noticed a mutation in the *wzc* of the *cps* gene cluster. Without a regulator how the *cps* gene cluster is regulated and enhances capsular polysaccharide synthesis remains a mystery.

The phylogenetic study revealed that all isolates were evolutionary depicted into four subgroups based on highly similar homology (Feng et al., 2021; Letunic and Bork, 2021), where, two hypervirulent isolates were originated from different sub-groups.

Still, this study has some limitations as we collected isolates from a single hospital over a short period. Therefore, a large-scale sample collection from different medical institutions is needed for further confirmation. Besides, this study remained to be investigated through mutant phenotypic experiments in wild-type cKp, and the virulence of isolates can be further confirmed through a mouse model.

In conclusion, we performed molecular epidemiology, phenotypic experiments, and comparative genomics of clinical MDR-hvKp isolates. This study discovered two MDR-hvKp isolates that showed hypervirulence and hyper-mucoid phenotype without both *rmpA* and *rmpA2* and hypervirulent defined genes. It challenged the existing knowledge to detect the hvKps. Hence, a large-scale genome-wide surveillance of MDR-hvKp isolates might meet this challenge. Before that, it is crucial to improve in-hospital ward monitoring to avoid nosocomial infections.

## Data availability statement

The datasets presented in this study can be found in online repositories. The names of the repository/repositories and accession number(s) can be found in the article/Supplementary material.

## Ethics statement

All isolates in this study were collected during the bacteriological analysis in the clinical microbiology laboratory of a public hospital, and patients were treated anonymously; therefore, ethical approval was not required. Under national law and institutional requirements, written informed consent was also not required.

## Author contributions

MRA and ZH designed the project and analyzed the data. MRA performed the experiments. YY, YD, and HL collected isolates. MRA wrote the manuscript. YL and BS supervised the project and obtained funding and critically revised the manuscript. All authors contributed to the article and approved the submitted version.

## Footnotes

^1^http://bigsdb.web.pasteur.fr

^2^https://github.com/tseemann/snippy

^3^https://www.graphpad.com/
