# Supplementary material for: Prevalence of multidrug-resistant hypervirulent Klebsiella pneumoniae without defined hypervirulent biomarkers in Anhui, China: a new dimension of hypervirulence
Source: Front Microbiol. 2023 Oct 5;14:1247091. doi: 10.3389/fmicb.2023.1247091 (PMC10585048; doi:10.3389/fmicb.2023.1247091)
Supplement: Supplementary file 1 [file Table_1.docx]

**Supplementary Tables**

**Prevalence of multidrug-resistant hypervirulent *Klebsiella pneumoniae* without defined hypervirulent biomarkers in Anhui, China: A new dimension of hypervirulence**

Md Roushan Ali^1^, Yu Yang^2^, Yuanyuan Dai^3^, Huaiwei Lu^3^, Zhien He^1*^, Yujie Li^1*^, and Baolin Sun^1*^

^1^Department of Oncology, The First Affiliated Hospital of USTC, Division of Life Sciences and Medicine, University of Science and Technology of China, Hefei, Anhui, China

^2^Department of Emergency Medicine, The Affiliated Provincial Hospital of Anhui Medical University, Hefei, Anhui, China

^3^Department of Clinical Laboratory, The First Affiliated Hospital of USTC, Division of Life Sciences and Medicine, University of Science and Technology of China, Hefei, Anhui, China

*Address correspondence to Zhien He ([zhienhe@mail.ustc.edu.cn](mailto:zhienhe@mail.ustc.edu.cn)),

Yujie Li ( [lyj2020@ustc.edu.cn](mailto:lyj2020@ustc.edu.cn) ) Baolin Sun ( [sunb@ustc.edu.cn](mailto:sunb@ustc.edu.cn))

| **Isolates Name** | **Collection date** | **Source** | **Patient age (Years)** | **Gender** | **Disease diagnosis** |
| --- | --- | --- | --- | --- | --- |
| 22112725 | 2022.11.27 | sputum | 85 | female | Chronic kidney disease |
| 22112805 | 2022.11.28 | sputum | 29 | male | Fracture |
| 22112350 | 2022.11.23 | sputum | 64 | male | Hepatolithiasis |
| 22122315 | 2022.12.23 | sputum | 92 | male | Chronic bronchitis |
| 22121932 | 2022.12.19 | sputum | 29 | male | Femoral shaft fracture |
| X22121850 | 2022.12.18 | blood | 69 | female | Coronary heart disease |
| 22122131 | 2022.12.21 | sputum | 71 | male | Breath difficulty |
| 22122138 | 2022.12.21 | sputum | 29 | male | Femoral shaft fracture |
| 22111436 | 2022.11.14 | sputum | 64 | male | Hepatolithiasis |
| 22111439 | 2022.11.14 | sputum | 81 | male | Infection |
| 22091569 | 2022.09.15 | sputum | 93 | male | Lung infection |
| 22111605 | 2022.11.16 | sputum | 50 | female | Head trauma |
| 22111648 | 2022.11.16 | sputum | 64 | male | Hepatolithiasis |
| 22091650 | 2022.09.16 | sputum | 57 | male | Fever |
| 22111719 | 2022.11.17 | sputum | 48 | male | Lung abscess |
| 22111788 | 2022.11.17 | sputum | 82 | female | Dizziness |
| 22111913 | 2022.11.19 | sputum | 90 | male | Chronic obstructive pulmonary disease |
| 22100407 | 2022.10.04 | sputum | 97 | male | Chronic obstructive pulmonary disease |
| 22090146 | 2022.09.01 | sputum | 70 | male | Benign tumors of the gums |
| 22110212 | 2022.11.02 | sputum | 83 | female | Head trauma |
| 22112203 | 2022.11.22 | sputum | 92 | male | Lung infection |
| X22083021 | 2022.08.30 | blood | 64 | male | Severe pancreatitis |
| X22083165 | 2022.08.31 | blood | 64 | male | Severe pancreatitis |

**Table S1:** Patient information of MDR-hvKp clinical isolates in this study

**Table S3:** MLST and K-typing

| **Isolates** | *infB* | *phoE* | *pgi* | *tonB* | *mdh* | *gapA* | *rpoB* | **MLST** | *wzi* | **K-typing** |
| --- | --- | --- | --- | --- | --- | --- | --- | --- | --- | --- |
| 22112725 | 3 | 1 | 1 | 4 | 1 | 3 | 1 | 11 | 64 | KL64 |
| 22112805 | 3 | 1 | 1 | 4 | 1 | 3 | 1 | 11 | 64 | KL64 |
| 22112350 | 1 | 9 | 1 | 12 | 1 | 2 | 4 | 23 | 1 | KL1 |
| 22122315 | 1 | 1 | 1 | 1 | 1 | 1 | 1 | 15 | 19 | KL19 |
| 22121932 | 3 | 1 | 1 | 4 | 1 | 3 | 1 | 11 | 64 | KL64 |
| X22121850 | 3 | 1 | 1 | 4 | 1 | 3 | 1 | 11 | 64 | KL64 |
| 22122131 | 3 | 1 | 1 | 4 | 1 | 3 | 1 | 11 | 64 | KL64 |
| 22122138 | 3 | 1 | 1 | 4 | 1 | 3 | 1 | 11 | 64 | KL64 |
| 22111436 | 1 | 9 | 1 | 12 | 1 | 2 | 4 | 23 | 1 | KL1 |
| 22111439 | 1 | 9 | 1 | 12 | 1 | 2 | 4 | 23 | 1 | KL1 |
| 22091569 | 1 | 1 | 52 | 7 | 2 | 4 | 1 | 307 | 173 | KL102 |
| 22111605 | 3 | 1 | 1 | 4 | 1 | 3 | 1 | 11 | 209 | KL47 |
| 22111648 | 1 | 9 | 1 | 12 | 1 | 2 | 4 | 23 | 1 | KL1 |
| 22091650 | 4 | 1 | 1 | 27 | 2 | 9 | 1 | 86 | 2 | KL2 |
| 22111719 | 4 | 7 | 1 | 38 | 6 | **3** | 4 | 147 | 64 | KL64 |
| 22111788 | 1 | 10 | 37 | 9 | 1 | 2 | 1 | 3132 | 267 | KL24 |
| 22111913 | 1 | 9 | 1 | 12 | 1 | 2 | 4 | 23 | 1 | KL1 |
| 22090146 | 1 | 1 | 52 | 7 | 2 | 4 | 1 | 307 | 173 | KL102 |
| 22110212 | 4 | 7 | 1 | 4 | 6 | 3 | 4 | 273 | 174 | KL74 |
| 22112203 | 1 | 7 | 1 | 12 | 1 | 2 | 1 | 485 | 555 | NA |
| X22083021 | 3 | 1 | 1 | 4 | 1 | 3 | 1 | 11 | 64 | KL64 |
| X22083165 | 3 | 1 | 1 | 4 | 1 | 3 | 1 | 11 | 64 | KL64 |

***Note:***

*Isolate 22100407 was excluded as it belonged to Klebsiella quasipneumoniae*

*NA: Not Available*

**Table S4:** Hypervirulence of clinical MDR-hvKp in *G. mellonella* infection model

| **Isolates** | **12 h survival**  **rate** (%mean±SD) | **24 h survival**  **rate**  (%mean±SD) | **36 h survival**  **rate**  (%mean±SD) | **48 h survival**  **rate**  (%mean±SD) | **60 h survival**  **rate** (%mean±SD) | **72 h survival**  **rate**  (%mean±SD) |
| --- | --- | --- | --- | --- | --- | --- |
| **22112725** | 100 ±0 | 70±10 | 53.34±15.27 | 33.34±20.82 | 23.33±11.55 | 10±10 |
| **22112805** | 96.67±5.78 | 56.67±30.56 | 40±36.05 | 30±26.46 | 26.67±20.82 | 10±10 |
| **22112350** | 40±34.64 | 33.34±32.14 | 0±0 | 0±0 | 0±0 | 0±0 |
| **22122315** | 0±0 | 0±0 | 0±0 | 0±0 | 0±0 | 0±0 |
| **22121932** | 100±0 | 50±10 | 40±10 | 30±0 | 26.67±5.78 | 23.34±5.78 |
| **X22121850** | 33.34±25.17 | 16.67±11.54 | 0±0 | 0±0 | 0±0 | 0±0 |
| **22122131** | 40±10 | 10±0 | 10±0 | 0±0 | 0±0 | 0±0 |
| **22122138** | 70±10 | 43.37±5.78 | 20±10 | 0±0 | 0±0 | 0±0 |
| **22111436** | 80±17.32 | 30±26.45 | 23.34±15.27 | 13.34±15.27 | 13.34±15.27 | 10±10 |
| **22111439** | 76.67±15.27 | 36.67±28.87 | 26.67±20.82 | 20±10 | 16.67±5.78 | 10±10 |
| **22091569** | 86.67±5.78 | 63.34±5.78 | 36.67±25.17 | 20±20 | 13.34±11.55 | 10±10 |
| **22111605** | 26.67±5.78 | 0±0 | 0±0 | 0±0 | 0±0 | 0±0 |
| **22111648** | 76.67±11.54 | 30±0 | 20±0 | 16.67±5.78 | 13.34±11.55 | 6.67±5.78 |
| **22091650** | 0±0 | 0±0 | 0±0 | 0±0 | 0±0 | 0±0 |
| **22111719** | 56.67±15.27 | 16.67±15.27 | 0±0 | 0±0 | 0±0 | 0±0 |
| **22111788** | 43.34±23.09 | 23.34±5.78 | 13.34±11.55 | 6.67±11.55 | 3.34±5.78 | 0±0 |
| **22111913** | 96.67±5.78 | 63.34±25.17 | 43.34±15.27 | 33.34±23.09 | 26.67±11.55 | 23.34±5.78 |
| **22100407** | 56.67±11.54 | 26.67±20.82 | 26.67±20.82 | 23.34±25.16 | 20±20 | 20±20 |
| **22090146** | 100±0 | 80±26.46 | 73.34±28.867 | 43.34±30.55 | 30±20 | 10±10 |
| **22110212** | 76.67±25.17 | 30±17.32 | 23.34±11.55 | 16.67±5.78 | 16.67±5.78 | 13.34±11.55 |
| **22112203** | 100±0 | 80±0 | 73.34±11.55 | 46.67±15.27 | 43.34±28 | 30±10 |
| **X22083021** | 36.67±5.78 | 0±0 | 0±0 | 0±0 | 0±0 | 0±0 |
| **X22083165** | 60±10 | 26.67±15.27 | 0±0 | 0±0 | 0±0 | 0±0 |
| **NTUH-K2044** | 83.34±11.55 | 70±5 | 70±5 | 60±4 | 50±10 | 40±10 |

*Note: ‘h’ indicates hour*

**Table S5:** Common missense mutation in hypervirulent isolate 22122315 compared to previous ST15 isolates (Zhao et al., 2022).

| **Missense mutant gene/locus** | **Effects** | **Functional products** |
| --- | --- | --- |
| *ybiU* | Asn352Lys (1056T>A) | putative protein YbiU |
| *glpD* | Asp390Gly (1169A>G) | Aerobic glycerol-3-phosphate dehydrogenase |
| *ygiF* | Ile39Asn (116T>A) | Inorganic triphosphatase |
| *mlaB* | Gln4His (12G>T) | Intermembrane phospholipid transport system binding protein MlaB |
| *sufB* | Cys448Tyr (1343G>A) | FeS cluster assembly protein SufB |
| *arnB* | His50Asn (148C>A) | UDP-4-amino-4-deoxy-L-arabinose--oxoglutarate aminotransferase |
| *rhlE_2* | Lys537Arg (1610A>G) | ATP-dependent RNA helicase RhlE |
| *srlE* | Ile6Val (16A>G) | PTS system glucitol/sorbitol-specific EIIB component |
| *gltB* | Thr581Ser (1741A>T) | Glutamate synthase [NADPH] large chain |
| *acrR_1* | Ile62Asn (185T>A) | HTH-type transcriptional regulator AcrR |
| *hscB* | Ser73Leu (218C>T) | Co-chaperone protein HscB |
| *dmlR_1* | Asn78Lys (234T>A) | HTH-type transcriptional regulator DmlR |
| *mdtJ* | Ile88Thr (263T>C) | Spermidine export protein MdtJ |
| *hexR_2* | Gly10Glu (29G>A) | HTH-type transcriptional regulator HexR |
| *cdhR* | Gly107Cys (319G>T) | HTH-type transcriptional regulator CdhR |
| *nodD2_1* | Pro112Thr (334C>A) | Nodulation protein D2 |
| *nlpI* | Asp120Tyr (358G>T) | Lipoprotein NlpI |
| *aceF* | Val128Ala (383T>C) | Dihydrolipoyllysine-residue acetyltransferase component of pyruvate dehydrogenase complex |
| *pulD* | Phe13Leu (39T>A) | Secretin PulD |
| *ogl* | Asn135Asp (403A>G) | Oligogalacturonate lyase |
| *ynfE* | Leu139Pro (416T>C) | Putative dimethyl sulfoxide reductase chain YnfE |
| *nagA* | Ile15Asn (44T>A) | N-acetylglucosamine-6-phosphate deacetylase |
| *pobA* | Asp153Val (458A>T) | p-hydroxybenzoate hydroxylase |
| *xynB* | Gly189Glu (566G>A) | Beta-xylosidase |
| *astD_3* | Thr223Asn (668C>A) | N-succinylglutamate 5-semialdehyde dehydrogenase |
| *pcaF* | Arg238Leu (713G>T) | Beta-ketoadipyl-CoA thiolase |
| *dmoA* | Glu300Asp (900A>T) | Dimethyl-sulfide monooxygenase |
| *dadX* | Pro319Ala (955C>G) | Alanine racemase, catabolic |
| *mdtG* | stop_lost& splice_region_variant_Gln382Terext*? | Multidrug resistance protein MdtG |
| *FEBNDAKP_03202* | Lys377Ile (1130A>T) | hypothetical protein |
| *FEBNDAKP_01342* | Lys93Arg (278A>G) | hypothetical protein |
| *FEBNDAKP_03262* | Ser108Ile (323G>T) | hypothetical protein |
| *FEBNDAKP_03237* | His157Tyr (469C>T) | hypothetical protein |
| *FEBNDAKP_02753* | Gly200Cys (598G>T) | hypothetical protein |
| *FEBNDAKP_03429* | Ile31Asn (92T>A) | hypothetical protein |
| *FEBNDAKP_03184* | Asn695Thr (2084A>C) | Putative tyrosine-protein kinase in *cps* region |

**Table S6:** Common missense mutation in hypervirulent isolate 22091569 compared to previous ST307 isolates (He et al., 2022).

| **Missense mutant gene/locus** | **Effects** | **Functional products** |
| --- | --- | --- |
| *hflK* | Ala366Val (1097C>T) | Modulator of FtsH protease HflK |
| *astD_1* | Ala414Val (1241C>T) | N-succinylglutamate 5-semialdehyde dehydrogenase |
| *sppA* | Gly520Ala (1559G>C) | Protease 4 |
| *degQ* | Val56Leu (166G>T) | Periplasmic pH-dependent serine endoprotease DegQ |
| *oxyR_5* | Lys7Glu (19G>A>G) | Hydrogen peroxide-inducible genes activator |
| *ribD* | Ala8Thr (22G>A) | Riboflavin biosynthesis protein RibD |
| *srlB_2* | Ala92Glu (275C>A) | PTS system glucitol/sorbitol-specific EIIA component |
| *nuoF* | Arg16Ser (46C>A) | NADH-quinone oxidoreductase subunit F |
| *mhpC* | Ala160Val (479C>T) | 2-hydroxy-6-oxononadienedioate/2-hydroxy-6- oxononatrienedioate hydrolase |
| *ulaF* | Ala190Ser (568G>T) | L-ribulose-5-phosphate 4-epimerase UlaF |
| *dnaJ* | Asp196Glu (588T>A) | Chaperone protein DnaJ |
| *fhuC_2* | Gln219Pro (656A>C) | Iron (3+)-hydroxamate import ATP-binding protein FhuC |
| *secE* | Val24Leu (70G>T) | Protein translocase subunit SecE |
| *EOFMAFIB_00699* | Arg396Cys (1186C>T) | hypothetical protein |
| *EOFMAFIB_00523* | Asn60Ile (179A>T), | hypothetical protein |
| *EOFMAFIB_00116* | Ala104Ser (310G>T) | hypothetical protein |
| *EOFMAFIB_00796* | Asn240Ile (719A>T) | hypothetical protein |
| *EOFMAFIB_02276* | Pro644Thr (1930C>A) | putative tyrosine-protein kinase in *cps* region |

**References**

He, Z., Xu, W., Zhao, H., Li, W., Dai, Y., Lu, H., Zhao, L., Zhang, C., Li, Y., Sun, B., 2022. Epidemiological characteristics an outbreak of ST11 multidrug-resistant and hypervirulent Klebsiella pneumoniae in Anhui, China. Front. Microbiol. 13. https://doi.org/10.3389/fmicb.2022.996753

Zhao, H., He, Z., Li, Y., Sun, B., 2022. Epidemiology of carbapenem-resistant Klebsiella pneumoniae ST15 of producing KPC-2, SHV-106 and CTX-M-15 in Anhui, China. BMC Microbiol. 22. https://doi.org/10.1186/s12866-022-02672-1
