# Supplementary material for: Prevalence of multidrug-resistant hypervirulent Klebsiella pneumoniae without defined hypervirulent biomarkers in Anhui, China: a new dimension of hypervirulence
Source: Front Microbiol. 2023 Oct 5;14:1247091. doi: 10.3389/fmicb.2023.1247091 (PMC10585048; doi:10.3389/fmicb.2023.1247091)
Supplement: Supplementary file 3 [file Data_Sheet_1.docx]

**Supplementary Figures**

**Prevalence of multidrug-resistant hypervirulent *Klebsiella pneumoniae* without defined hypervirulent biomarkers in Anhui, China: A new dimension of hypervirulence**

Md Roushan Ali^1^, Yu Yang^2^, Yuanyuan Dai^3^, Huaiwei Lu^3^, Zhien He^1*^, Yujie Li^1*^, and Baolin Sun^1*^

^1^Department of Oncology, The First Affiliated Hospital of USTC, Division of Life Sciences and Medicine, University of Science and Technology of China, Hefei, Anhui, China

^2^Department of Emergency Medicine, The Affiliated Provincial Hospital of Anhui Medical University, Hefei, Anhui, China

^3^Department of Clinical Laboratory, The First Affiliated Hospital of USTC, Division of Life Sciences and Medicine, University of Science and Technology of China, Hefei, Anhui, China

*Address correspondence to Zhien He ([zhienhe@mail.ustc.edu.cn](mailto:zhienhe@mail.ustc.edu.cn)),

Yujie Li ( [lyj2020@ustc.edu.cn](mailto:lyj2020@ustc.edu.cn) ) Baolin Sun ( [sunb@ustc.edu.cn](mailto:sunb@ustc.edu.cn))


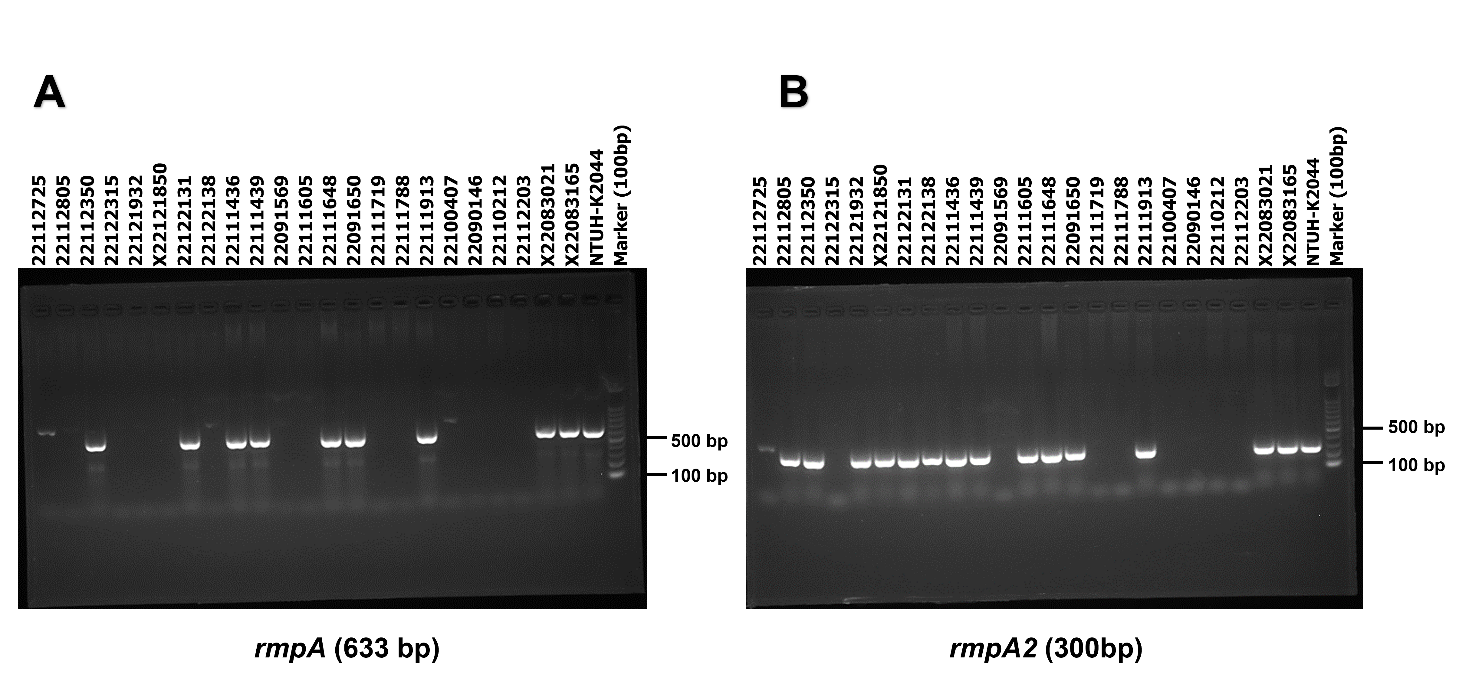


**Fig S1**: Detection of regulator of mucoid phenotype gene, *rmpA* and *rmpA2* in MDR-hvKp clinical isolates (A-B). (A) Presence of *rmpA* gene (633 bp) in MDR-hvKp clinical isolates, (B) Presence of *rmpA2* gene (300 bp) in MDR-hvKp clinical isolates


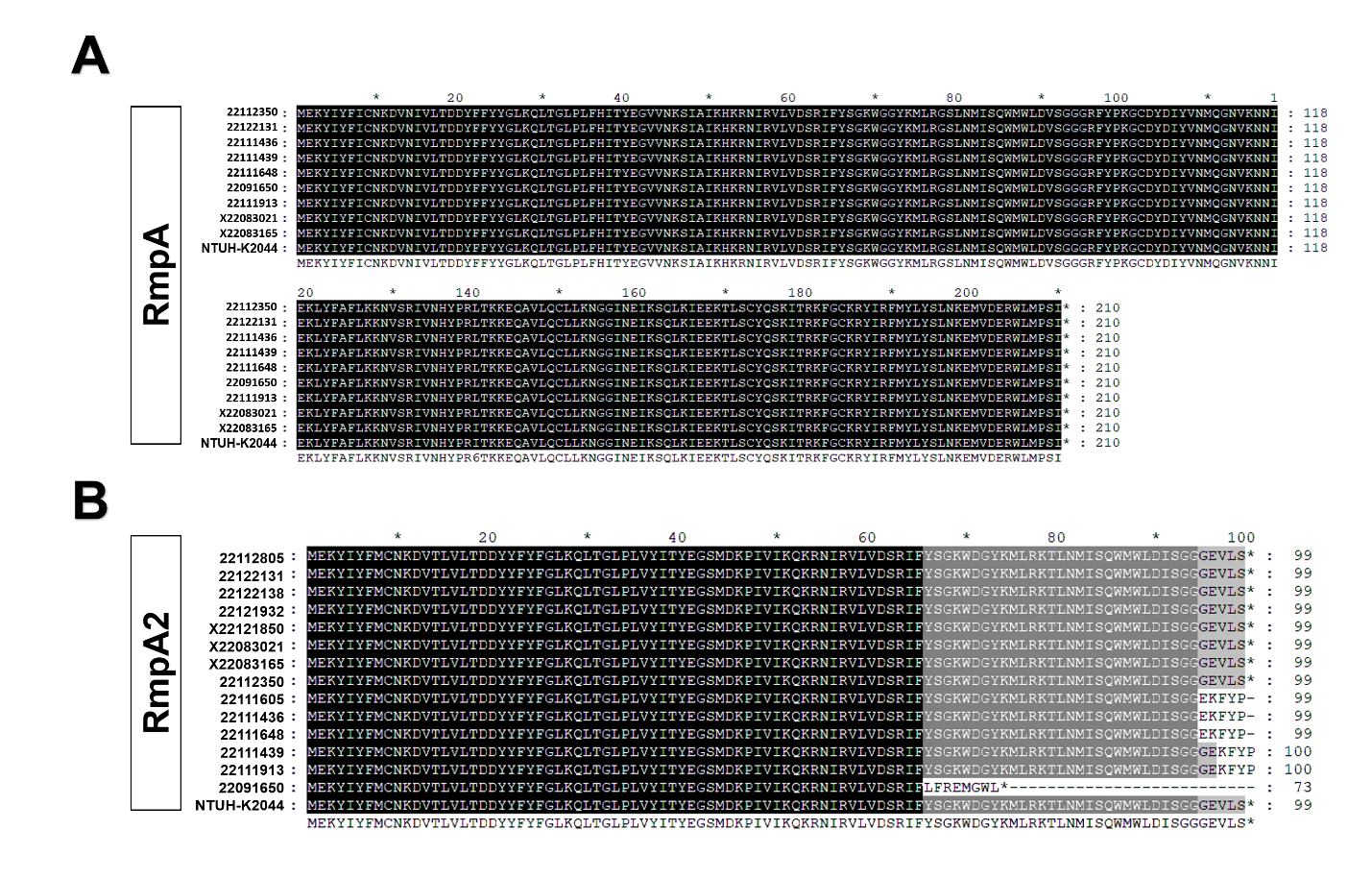


**Fig. S2**: Alignment of protein sequences of RmpA and RmpA2 from MDR-hvKp clinical isolates using GeneDoc. (A) Alignment of RmpA protein sequences from different *rmpA* positive MDR-hvKp isolates (B) Alignment of RmpA2 protein sequences from different *rmpA2* positive MDR-hvKp isolates.


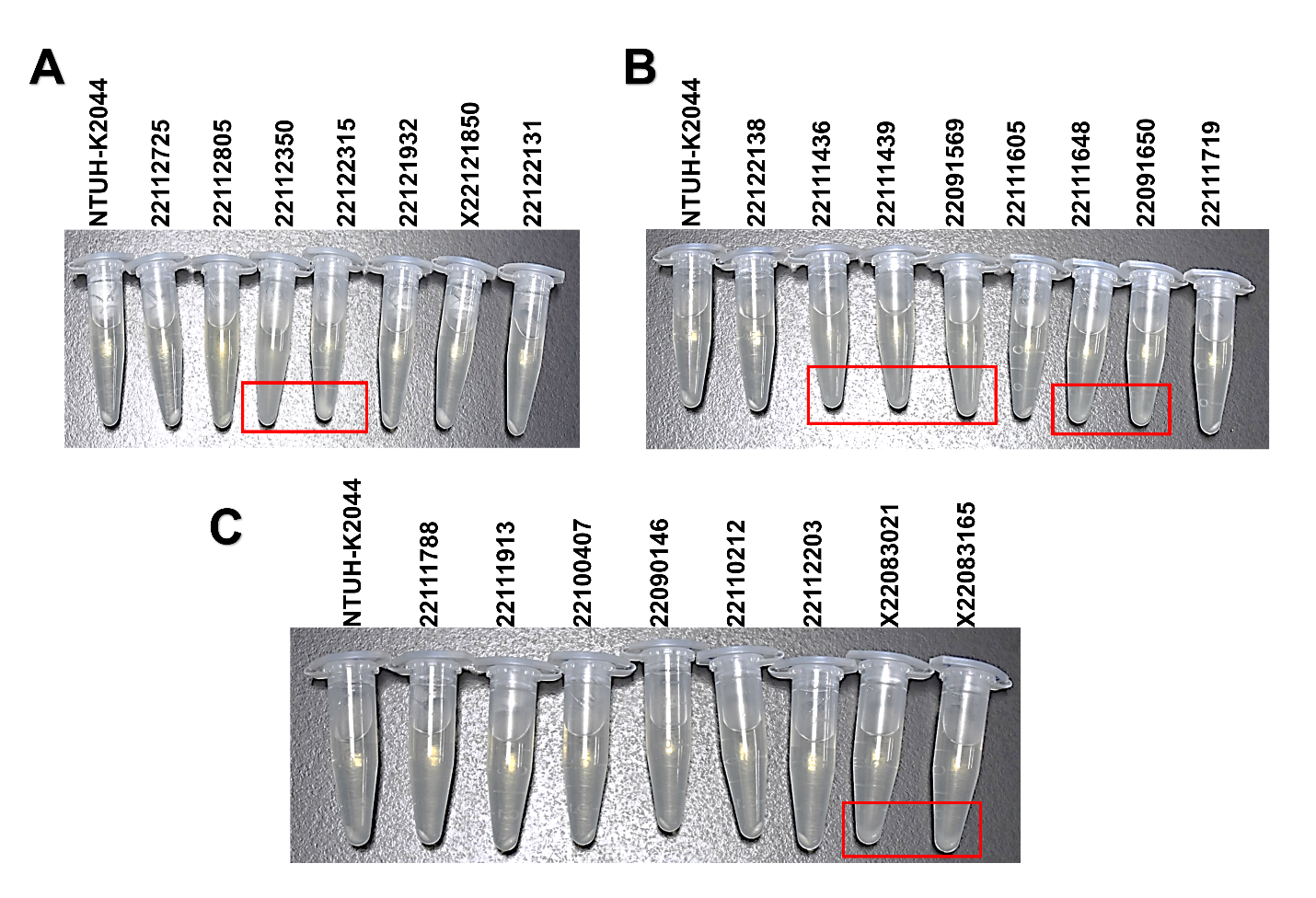


**Fig. S3**: Screening of hyper mucoid isolates from the MDR-hvKp clinical isolates (A-C). Mucoid phenotype was determined by the low-speed centrifugation (2,350 × g for 5) of the overnight culture for 5 min, compared to control NTUH-K2044.


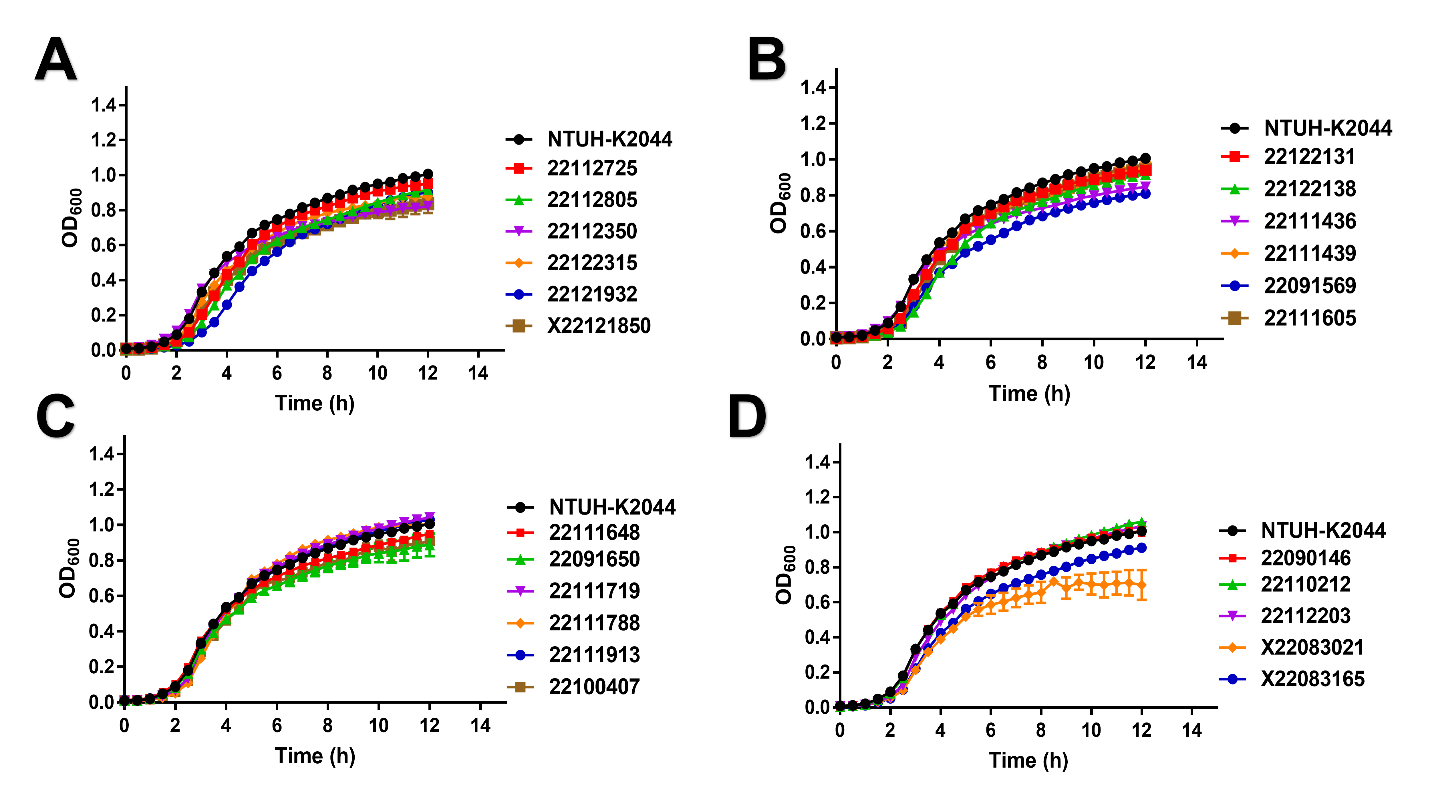


**Fig. S4**: Growth curve of clinical isolates (A-D). Growth curves were evaluated compared to control (NTUH-K2044)
